# Personalized Medicine and Cancer

**DOI:** 10.3390/jpm2010001

**Published:** 2012-01-30

**Authors:** Mukesh Verma

**Affiliations:** Epidemiology and Genetics Research Program (EGRP), Division of Cancer Control and Population Sciences, National Cancer Institute (NCI), National Institutes of Health (NIH), 6130 Executive Boulevard, Rockville, MD 20852, USA; E-Mail: vermam@mail.nih.gov; Tel.: +1-301-594-7344; Fax: +1-301-435-5477

**Keywords:** cancer, diagnosis, epigenome, genome, metabolome, personalized medicine, outcome, proteome, survival, treatment

## Abstract

Cancer is one of the leading causes of death in the United States, and more than 1.5 million new cases and more than 0.5 million deaths were reported during 2010 in the United States alone. Following completion of the sequencing of the human genome, substantial progress has been made in characterizing the human epigenome, proteome, and metabolome; a better understanding of pharmacogenomics has been developed, and the potential for customizing health care for the individual has grown tremendously. Recently, personalized medicine has mainly involved the systematic use of genetic or other information about an individual patient to select or optimize that patient’s preventative and therapeutic care. Molecular profiling in healthy and cancer patient samples may allow for a greater degree of personalized medicine than is currently available. Information about a patient’s proteinaceous, genetic, and metabolic profile could be used to tailor medical care to that individual’s needs. A key attribute of this medical model is the development of companion diagnostics, whereby molecular assays that measure levels of proteins, genes, or specific mutations are used to provide a specific therapy for an individual’s condition by stratifying disease status, selecting the proper medication, and tailoring dosages to that patient’s specific needs. Additionally, such methods can be used to assess a patient’s risk factors for a number of conditions and to tailor individual preventative treatments. Recent advances, challenges, and future perspectives of personalized medicine in cancer are discussed.

## 1. Introduction: Personalized Medicine and Cancer

Although cancer incidence and prevalence are increasing at an alarming rate, progress in treatment has been slow, and treatment benefits are measured in weeks to months. Traditionally, patient care is given by physicians based on pathological examination, symptoms of the disease, and history of medications. Following advancements in diagnostic science and early detection markers, a number of cancer types can be detected before pathological symptoms develop. These markers are biochemical, epigenetic, genetic, imaging, metabolomic, and proteomic. Technologies can be used to detect these markers in clinical samples with an option of multiplexing. Use of more than one marker in the same sample generally increases the sensitivity and specificity of cancer detection and helps a physician to diagnose early and accurately. This information is of great significance because individual specific treatment regimens can be designed based on the presence and stage of cancer as concluded from profiles of markers discussed above. Pathological diagnosis is still gold standard in clinical practice; however, molecular diagnosis with additional information may be different from pathological diagnosis.

Genetic aberrations, either somatic or hereditary, may lead to cancer. Hereditary cancers, which are a major part of medical genetics, can be understood by following cancer genetics. Familial cancers cover only 10–15% of total cancers, and the remaining cancers are influenced by environmental factors, infections, and lifestyle. This information helps scientists determine the risk of cancer development in an individual’s lifetime [1]. However, there are only a few cancer-disposing syndromes in which an allele is segregated in an autosomal-dominant fashion, thereby contributing to a high risk of cancer development. Furthermore, non-genetic factors contribute to mutations or other genetic changes. Cancer also has been observed to develop in individuals who have no family history of cancer.

Along with genetic variations in tumors *per se*, inherited genetic variants in genes that metabolize and process drugs also influence response to treatment. These variants may increase the toxicity of specific drugs. This knowledge has enabled the development of the science of “pharmacogenomics,” which identifies individuals who, based on their genotype information, will respond to a specific therapy [2]. The goal of personalized medicine is to use the right drug at the right dose, with minimal or no toxicity, for the right patient at the right time. This article discusses the state of the art of this science using the example of cancer.

## 2. Why Personalized Medicine Is Needed

Although DNA from different cells is the same, genes coding in one organ (and their cells) behave differently than genes in other organs. In cancer, different tumors may have the same DNA, but the gene expression pattern is different in different tumor types. Technologies such as gene-expression microarray allow us to examine the gene expression profile of hundreds of genes at a time and to distinguish a cancer-associated gene expression profile from normal profiling. For decades, standard medical care has been guided by cohort-based epidemiological studies in which the genetic variability of individuals is not accounted for and most of the conclusions are based at the population level [3]. Modern personalized medicine takes into account an individual’s genetic makeup and disease history before a treatment regimen is generated. This is in contrast to traditional personalized medicine, in which care is based on a patient’s family history, social circumstances, environment, and lifestyle.

Modern personalized medicine is based on targeted therapy. In targeted therapy, it is essential that information about the altered pathway and the components leading to cancer are available. For example, Herceptin is used in female breast cancer patients who express higher levels of HER-2. Gleevec is prescribed in chronic myleloid leukemias to inhibit tyrosine kinase. In these patients, reciprocal translocation between chromosome 9 and chromosome 20 occurs, resulting in hyperactivation of abl-driven protein signaling. This point is explained in detail below.

## 3. The Contribution of “Omics” to Personalized Medicine in Cancer

The traditional approach to personalized medicine has been a “reactive” approach in which a doctor examines a patient, diagnoses the disease based on symptoms, and then prescribes medicine. In contrast, in modern treatment, a doctor evaluates the patient’s genetic background and family history first and then prescribes treatment. We now know more about genomic variations (copy number changes, deletions, mutations, single nucleotide polymorphisms) and the association of these variations with different cancers. These association studies help in determining who is at high risk of developing cancer. It is understood and hoped that cancer genomics aims to advance personalized medicine through DNA sequencing and the analysis of cancer cells from patients to find new genetic alterations associated with specific cancers. Constructing a comprehensive catalog of the key genomic changes in many major types and subtypes of cancer will support advances in developing more effective ways to diagnose, treat, and prevent cancer. Cancer is a disease of the genome. As more is learned about specific tumor types, it strengthens the conclusion that each tumor has its own set of genetic changes. Understanding the genetic changes and gene expression profiles that are in cancer cells is leading to more effective treatment strategies that are tailored to the genetic profile of each individual patient’s cancer. At the National Institutes of Health (NIH), the Cancer Genome Atlas (TCGA) project explores information and resources to improve our understanding of cancer genomics, the importance of tumor samples in genomic research, and the role of cancer genomics in personalized medicine.

In addition to genomic information, proteomic information also contributes to personalized medicine. In the Human Proteome Project, a profile of all of the peptides and proteins present in a clinically healthy person was determined and compared to the peptide and protein profile from a cancer patient. By characterizing all 21,000 genes of the known genome, the Human Proteome Project has generated a map of the protein-based molecular architecture of the human body and become a resource to help elucidate biological and molecular functions and advance the diagnosis and treatment of diseases. Although the capability of functions is coded in genes, the actual function is accomplished by proteins. Therefore, understanding protein profiles and their expression in normal and cancer states is essential. Most of the drugs approved by the U.S. Food and Drug Administration (FDA) are directed toward proteins. In fact, at early stages, those assays that were approved by FDA for cancer detection and diagnosis were protein based (immunohistochemistry). The major pathways in cancer development (such as the receptor kinase pathway, m-Tor pathway, MAP kinase pathway, apoptosis, EGFR pathway, tyrosine kinase pathway, Notch pathway) and their interactions (signal transduction) are based on protein interactions. Although the cancer process is initiated by mutation, its expression is mediated by proteins and enzyme-mediated signal transduction pathways.  

The International Human Epigenome Consortium (IHEC) coordinates the production of reference maps of the human epigenome for key cellular states relevant to health and disease, including cancer . To achieve substantial coverage of the human epigenome, the IHEC set the ambitious goal of deciphering at least 1,000 epigenomes within the next 7–10 years. The plan is to produce high-resolution maps of informative histone modifications, high-resolution DNA methylation maps, landmark maps for the transcription start sites of all protein-coding genes, the entire catalogue and expression patterns of non-coding and small RNAs, and comparative analysis of epigenome maps of model organisms relevant to human health and disease. Surveys of individuals, pedigrees, and genetically identical twins will be used to determine the relationship between genetic and epigenetic variation worldwide. NIH Roadmap Epigenomics is another program that provides epigenomic maps as reference standards.

Metabolomics, a new addition to the field of personalized medicine, is the study of low molecular weight molecules or metabolites found within cells and biological systems. The metabolome is a measure of the output of biological pathways and, as such, is often considered more representative of the functional state of a cell than other “omics” measures such as genomics or proteomics. As an example, acetoamide (paracetamol)-treated patients are followed for treatment response via metabolic profiling of their urine and blood. Pre- and post-dose analysis shows high p-cresol sulfate before treatment and low acetoamide sulfate to acetylamino glucuronide after treatment. Common technologies for measuring the metabolome include mass spectrometry (MS) and nuclear magnetic resonance (NMR) spectroscopy , which can measure hundreds to thousands of unique chemical entities. Despite early promise, challenges remain before the full potential of metabolomics can be realized. Existing metabolomics facilities are at capacity, with relatively few scientists who possess in-depth expertise in metabolomics and a dearth of training opportunities to provide that expertise. Some companies provide metabolomics services and limited standards; however, issues concerning cost, intellectual property rights, and limited profit incentives minimize their use in basic, clinical, and translational research.

## 4. Examples of Personalized Medicine in Different Cancers

The design of personalized health care is based on prevention or therapeutic approaches in conjunction with current knowledge of the cancer type [4]. Although personalized medicine has been used in a number of cancers, we have selected few cancers below where incidence and prevalence of cancer is high in US and more data is available compared to other cancers. Furthermore, all types of cancer cannot be covered in one article, so I have selected breast, colon, lung, prostate, myeloid neoplasia, leukemia and lymphoma below.

### 4.1. Breast Cancer

Based on mortality rate, breast cancer is the leading cancer in females. The factors contributing to breast cancer are genetic, environmental, and behavioral (diet, exercise, and lifestyle). Preventive approaches such as mammogram screening have been adopted by a large population. Screening for *BRCA1* and *BRCA2* mutations also is a common practice in clinics for women in different age groups and parity status. Song *et al.* discussed current and future personalized medicine approaches in breast cancer patients [5]. Because of differences in individuals’ genetic backgrounds and personal susceptibility to environmental and modifiable factors, interventions do not always succeed. Increasing evidence supports personal genomic susceptibility as the major factor in responding to intervention and prevention. The approach provided by these investigators includes behavior modification for high-risk subjects (primary prevention), early detection and extensive monitoring of genetically susceptible subjects and noninvasive treatment of early stage cancer cases (secondary prevention), and finally prophylactic and therapeutic intervention to slow disease progression (tertiary prevention). Based on the molecular characterization of breast cancer, individualized preventive strategies for personalized health care may be designed and implemented, although some controversies also exist which I have discussed at the end of this section. CYP2D6 (cytochrome P450, family 2, subfamily D, polypeptide 6) genotyping and its influence on breast cancer treatment by tamoxifen indicate the importance of personalized medicine in treating patients [6]. Tamoxifen is a standard treatment (endocrine therapy) for steroid receptor positive breast cancer patients. Cytochrome P450 activates tamoxifen and forms active metabolites 4-hydroxytamoxifen and endoxifen [7]. These metaboloites have two order of magnitude affinity towards the steroid receptor compared to tamoxifen. These compounds inhibit proliferation of cells. CYP2D6 has different variants and poor metabolizers and severely impaired CYP2D6 are suggested to be associated with high recurrence of breast cancer [8]. Thus genotyping of CYP2D6 before treatment may predict response to treatment. Intelligent clinical decision can be made about the option of choosing strong CYP2D6 inhibitors which may inactivate active metabolites. Because the pharmacogenomics based approaches use CYP2D6 genotyping to have an idea about personal metabolizer phenotype, ethical issues must be addressed in advance. Patients and their caregivers should be well informed about the treatment strategies [9]. Raloxifene becomes an alternative choice of treatment in CYP2D6 poor metabolizer patients [9]. Recommendations for broad CYP2D6 allele coverage and high-throughput MALDI-TOF MS/CAN (matrix-assisted laser desorption adsorption time-of-flight mass spectrometry/copy number assay) have been made by Schroth *et al.* [10] to reduce phenotypic misclassification. Erb-B2 expression based therapy of breast cancer has shown promising results in the field of personalized medicine [11,12]. Recent report, however, indicates that routine assessment of CYP2D6 should not be used as a guide for tamoxifen treatment and other factors should also be considered [13,14,15]. These investigators have suggested that aromatase inhibitors should not be administered to those patients who are pre- or permenopausal. Fleeman *et al.* [16] have suggested additional research on alleles other that CYP2D6 and identify patients who are responsive to treatment by tamoxifen. Norendoxifen, a metabolite of tamoxifen is considered a potential lead compound in therapeutics due to its inhibition properties of aromatase [17]. Other reports suggest that MammPrint and Oncotype DX are current diagnostic tools which are based on expression profiling and have promising results in personalized medicine [18,19,20]. Future “omics” research may also add valuable information in personalized treatment of breast cancer as omics approach, including genomics, epigenomics, transcriptomics, proteomics, Metabolomics, interactomics, brings powerful ability to screen cancer cells at different stages of disease development leading to novel therapeutic target identification and validation of known targets.

### 4.2. Colon Cancer

The genetics and epigenetics of colon cancer are well characterized, and biomarkers for the early detection of colon cancer are known. A number of common treatments for colon cancer are available (chemotherapy, radiation, and surgery) [21,22]. Furthermore, colonoscopy screening has helped in detecting this cancer when polyps are just beginning to form. A correlation of mutations, microsatellite instability, and hypermethylation in tumors from individual patients is being completed. The information from such experiments will help to identify subgroups that are likely and not likely to respond to a particular treatment regimen [4]. This will allow patients who are likely to benefit to receive optimal care and allow those who are unlikely to benefit to avoid unnecessary toxicity and costs. In general, when colon cancer is treated at an early stage, many patients survive at least 5 years after their diagnosis. If the colon cancer does not recur within 5 years, the disease is considered to be cured. Stage I, II, and III cancers are considered potentially curable. In most cases, stage IV cancer is not considered curable, although there are exceptions. One investigator has different opinion about this and according to this investigator 5 year survival should not be considered potentially curable because late recurrences are known to arise in colon cancer and other tumor entities as well and the 5 year survival is a rate decreasing with higher cancer stage (even in stages I–III).

It has also been observed that certain therapy does not work in colorectal cancer. For example, KRAS mutations, which cover about 40% of colorectal cancers, make the tumor unresponsive to anti-epidermal growth factor receptor therapy with cetuximab or panitumumab [23,24,25]. In terms of pharmacogenomics of colon cancer, Sarasqueta *et al.* [26] recently evaluated polymorphism in GSTP1, ERCC1, and ERCC2 (genes involved in the metabolism of oxaliplatin) and its correlation with the prediction of disease. In another study, Maxican patients treated with 5-flurouracil and folinic acid predicted reponse to treatment with the absence or presence of polymorphism in methylenetetrahydrofolate reductase (MTHFR) gene [27]. miRNA polymorphism has been demonstrated to be associated with response to treatment with 5-fluorouracil and irinotecan [28].

### 4.3. Lung Cancer

There are two main types of lung cancer: non-small cell lung cancer (NSCLC) and small cell lung cancer (SCLC). Small cell lung cancer makes up about 20% of all lung cancer cases. Cancer made up of both types is called mixed small cell/large cell cancer. If the cancer started somewhere else in the body and spread to the lungs, it is called metastatic cancer to the lung. Because of the heterogeneity of cells, it is extremely difficult to treat lung cancer. Regular treatment techniques, mainly surgical and chemotherapy, have been used to treat lung cancer. Based on recent data and understanding of the genetic basis of lung cancer, EGFR, K-ras, ALK, MET, CBL, and COX2 are being used as therapeutic targets [29]. Curran [30] recently demonstrated utilization of crizotinib in the treatment of NSCLC. Crizotinib is and inhibitor of anaplastic lymphoma kinase (ALK) and showed promising results. Other investigators have also observed benefits of using crizotinib for lung cancer treatment [31,32]. Erlotinib and EGFR mutated lung cancer has also provided significant clinical results [33]. FLEX trial has also demonstrated promising results [34]. Data from histopathological examination and the patient’s history also is considered in evaluating the state of the disease and its aggressiveness. Nyberg et al [35] studied association between SNPs and acute interstitial lung disease in Japanese population undergoing treatment with gefitinib. This research provided basis for further research. In Chinese population, ABCC1 polymorphism was found to be associated with lung cancer susceptibility in patients undergoing chemotherapy [36]. Genomic variations in EGFR and ERCC1 have also been correlated with drug response in small cell lung cancer patients [37,38].

### 4.4. Prostate Cancer

Prostate cancer is the leading cancer in men and the second leading cause of death due to cancer. High rates of this cancer are observed in older people. The main screening procedures used to detect prostate cancer are the digital rectal exam and prostate specific antigen (PSA) test. Because this cancer does not cause pain and takes several years to develop, physicians and patients are faced with the challenge of identifying optimal treatment strategies for localized prostate cancer, biochemically recurrent prostate cancer, and later-stage cancer. Three treatments are very common: chemotherapy and hormonal therapy, surgery, and radiation. Age-related changes, including metastatic disease, may affect all of these therapies and shift the risk-benefit ratio of these treatments [39]. New tools, such as the Comprehensive Geriatric Assessment, are being developed to better predict who will respond to therapy. Such tools also may help in estimating the remaining life expectancy of a specific prostate cancer patient. Audet-Walsh *et al.* [40] demonstrated association of several SRD5A1 (steroid 5-alpha reductase) and SRD5A2 variations as independent predictors of biochemical recurrence after radical prostatectomy in Caucasians and Asians. In another study, BCL2 polymorphism was found to be associated with adverse outcome in prostate carcinoma patients [41].

### 4.5. Myeloid Neoplasia

Abnormal genetic and epigenetic events contribute to the development of myeloid neoplasia. Most of these alterations have been localized in hematopoietic differentiation and cellular proliferation pathways [42]. A number of therapeutic agents have been developed to treat myeloid dysplasia. Attempts are being made to integrate pathological information with genomic information so that future directions in personalized genomics can be explored [43].**Lymphomas are closely related to lymphoid leukemias, which also originate in lymphocytes but typically involve only circulating blood and the bone marrow (where blood cells are generated by hematopoiesis) and usually do not form static tumors. There are many types of lymphomas and, in turn, lymphomas are a part of the broad group of diseases known as hematological neoplasms. Takahashi *et al*. [44] demonstrated CYP3A5 polymorphism on imatinib traugh concentration and clinical response among patients with chronic phase myeloid leukemia.

### 4.6. Lymphoma and Leukemia

Lymphoma is a cancer in the lymphatic cells of the immune system. It is present as a solid tumor of lymphoid cells. Similar to other cancers described in this article, research is being conducted to utilize the clinical characterization of lymphoma and integration of genomic information to identify patients who will benefit from the treatment. Lymphoma comprises mainly Hodgkin lymphoma and non-Hodgkin lymphoma, although at least 60 subtypes of lymphoma have been reported to date [45]. This cancer originates from lymph nodes but can affect other organs such as the bowel, bone, brain, and skin. Risk-stratification for all clinically identified subtypes has not been completed yet. Approaches for the stratification of lymphoma subtypes include refining clinical prognostic models for better risk stratification, use of high-throughput technology to identify biologic subtypes within pathologically similar diseases, “response-adapted” changes in therapy via imaging with [(18)F]fluoro-2-deoxy-d-glucose positron emission tomography (FDG-PET), and anti-idiotype vaccines. Lymphoma treatment is accomplished by chemotherapy, radiation therapy, and bone marrow transplantation.

An effective treatment for acute promyelocytic leukemia consists of identifying and developing the PML-RARA fusion gene and applying all-trans retinoic acid (ATRA) [46]. This investigation has led to the discovery of the bcr-abl fusion gene in chronic myelogenous leukemia and development of imatinib [47].

Genetics-based drug therapy does not always work efficiently. Erlotinib and crizotinib are other genetics-based drugs with minimum efficacy in different cancers [48]. The mechanism of action of these medications is based on apoptosis. The reason for developing apoptosis-based therapies is the advantage of killing cancer cells specifically with low or minimal toxicity. These drugs were not effective because the differentiation and proliferation pathways were not affected by these drugs. In an ideal situation, the drug should inhibit all of these pathways and stop the signaling steps. To attack the final steps in the apoptosis pathway and achieve better efficacy, human recombinant DNAse I-based drugs are being developed [49]. Polymorphisms in mismatch repair genes influence response to treatment and survival in large B cell lymphoma [50]. Vagace *et al.* [49] identified presence of numerous genetic variants that may have accounted for subacute methotrexate neurotoxicity in acute lymphoblastic leukemia. 

## 5. Challenges, Future Perspectives, and Conclusions

As personalized medicine becomes more popular and more commonplace, those who pay for the treatment are affected. For insurance companies, providing health care is more expensive when more tests are performed to diagnose a disease and when customized treatment is used. In the long term, personalized medicine will be beneficial because information about a person’s disease and responsiveness to different interventions and treatment will be helpful in developing disease-prevention approaches. Only 5% of all private health insurance companies cover genetic tests. This raises the issue of how successful personalized medicine can be in the United States under the current health care delivery system. Insurance companies calculate premiums based on expenses in large populations, whereas the cost of personalized medicine is calculated for much smaller numbers of individuals. For personalized medicine to succeed, large-population models must be revised. It will cost the payer less in the long term if a precise diagnosis is provided to avoid unnecessary and ineffective treatments, prevent adverse events, and deliver more effective targeted therapeutics. This also will help to promote the “pay for performance” concept and reduced health care costs. Ethical issues and genetic tests are further topics for consideration to implement personalized medicine and data should be collected and analyzed on these aspects.

Health care providers must develop tools to maintain up-to-date patient data and sophisticated decision-making support tools. Doctors and primary care physicians should do their jobs better by acquiring an educational background and hands-on experience in genomic and proteomic tests and their interpretation, developing decision-making tools, and creating service lines around prevention and wellness to replace revenues lost by traditional medical practice. When determining treatment, an oncologist needs to weigh not just the genes and biology of the cancer but the age, medical condition, lifestyle, and goals of each patient. Government should play an active role in approving personalized medicine tests quickly and provide incentives for using them. The Genomics and Personalized Medicine Act was introduced in the U.S. Congress and covers scientific barriers, adverse market pressures, and regulatory obstacles. Public education and communication about personalized medicine should be part of the outreach to the population at large. Furthermore, consumers should be protected from possible harm resulting from the premature translation of research findings, and the innovative and cost-effective application of discoveries that improve personalized medical care should be encouraged.

Currently, we see only a few successful examples of personalized medicine, such as the measurement of erbB2 and EGFR in breast and lung cancer patients before proper treatments are selected. Successful implementation of personalized medicine will require the infrastructure and technology to assay molecular analytes and collaboration between all stakeholders. In personalized medicine, the key task is to identify and validate key proteins, different expression patterns, and gene variants associated with disease or disease predisposition; and better genotype-phenotype relationship (as I discussed above about CYP2D6 polymorphism and breast cancer). Today’s biomarker is tomorrow’s theranostics. Theranostics is the term used to describe the proposed process of diagnostic therapy for individual patients—to test them for possible reaction to taking a new medication and to tailor a treatment for them based on the test results.

The required infrastructure also includes a high level of collaboration among specialists to integrate and make sensible conclusions from available data. Personalized medicine involves not only tailoring the right treatment/drug for the right person but also evaluating predisposition to disease, sometimes several years before a disease is fully developed (for example, before metastasis). Additional aspects of the infrastructure remain to be established before personalized medicine can be translated into practice.  Examples of genetics-based drug are ipilimumab and PLX4032 which showed improvement in survival of melanoma patients.

The diagnostic and therapeutic markets are expected to benefit from the advancement of personalized medicine. Furthermore, beyond core products and services, more consumer-oriented areas also are expected to benefit. These include markets such as nutrition and wellness, complementary and alternative medicine, nutraceuticals and organic care, health and exercise equipment and health clubs/fitness centers, telemedicine, electronic record data entry, and disease management services.

In the future, a “bench to bedside” approach will be followed that will be based on epidemiologic studies. These studies will test the newly discovered intervention from preclinical trials in first clinical trials. During this time, population studies and clinical studies will be designed to assess the prevalence, associations, interactions, sensitivity, specificity, and predictive value of genetic and non-genetic factors. Some specific issues concerning the future of personalized medicine in cancer are presented in Table 1. Some of the issues and their potential resolution are: matching advanced technologies (genomics, proteomics, epigenomics) with *in silico* techniques by validating these technologies in large number of samples; resolving tumor heterogeneity-associated problems in patient’s molecular profiles by making standards of profiling collecting from a large number of data sets collected from clinical samples with different stages of disease development; defining drug efficacy relevant genotype phenotype relationships by selecting well characterized genetic alterations in molecularly and pathologically characterized samples and studying the effects of drugs against these backgrounds; resolving tumor heterogeneity-associated problems by characterizing laser captured sample characterization; training medical staff in the application and interpretation of results from molecular profiles by providing facilities, education and training to the staff; reimbursing patients related to molecular profiling by adding funds in the system; resolving the risk of litigation in personalized medicine by discussion with practitioners, insurance and policy makers.

**Table 1:** Specific issues in personalized medicine and cancer.

Special Issues	Reference
Matching advanced technologies (genomics, proteomics, epigenomics) with *in silico* techniques	[2]
Resolving tumor heterogeneity-associated problems in patient’s molecular profiles	[2,12]
Defining drug efficacy relevant genotype phenotype relationships	[4]
Resolving tumor heterogeneity-associated problems	[21]
Training medical staff in the application and interpretation of results from molecular profiles	[12,22]
Reimbursing patients related to molecular profiling	[1]
Resolving the risk of litigation in personalized medicine	[1]

It is timely and important to ask: Are we at the point of being able to treat each patient uniquely based on the complete DNA structure of their cancer? The answer is no, we are not there yet. However, the field is evolving, and personalized medicine has much to offer toward improving cancer treatment for today and tomorrow.
